# A Probiotic Friend

**DOI:** 10.1128/mSphere.00856-21

**Published:** 2021-12-22

**Authors:** Silke Dubbert, Rudolf von Bünau

**Affiliations:** a Ardeypharm GmbH, Herdecke, Germany; UTMB

**Keywords:** colibactin, probiotics

## LETTER

We read with interest the publication by Nougayrède and coworkers who described that Escherichia coli strain Nissle 1917 (EcN) is mutagenic by means of its expressed colibactin (1). The contribution of this work on the biological activity of colibactin is of high significance and increases our knowledge on this enigmatic toxin. The use of mouse models is an important methodology to study the effect of colibactin *in vivo.* Nougayrède et al. provided evidence that EcN has genotoxic potential in the models they applied (1).

The topic of colibactin is of extreme importance to our company, as patient and consumer safety has our utmost priority: the use of probiotic products that contain EcN as an active ingredient should not have health risks. That has been the basis of our company’s strategy in the past, and it remains our focus in the present and future. For this reason, databases on drug side effects, such as EudraVigilance (the European database of suspected adverse drug reaction reports, www.adrreports.eu) are closely being monitored. So far, these show no evidence of a potential cancer risk from intake of EcN.

As this matter is of high concern to us, we had conducted genotoxicity tests that are compliant with FDA Recommendations (2) and OECD Guidelines (3, 4). These guidelines have been defined to detect genotoxicity of a given compound, and commercial companies are obliged to follow such guidelines. This included use of the Ames test, performed according to the original test protocol and additionally with slight modification for testing live EcN bacteria (5). No mutagenic or cytotoxic effect of live EcN or its metabolites were found with these tests (5). Needless to say, tests were performed according to good manufacturing practicing (GMP) standards with inclusion of all appropriate controls. These controls identified that the number of spontaneous revertants in the Ames test were in accordance with the frequency range reported in the literature and with in-house historical data. Compared to this control, the presence of EcN did not raise the number of revertant colonies of the Ames strains and it did not reduce the number of the Ames strain colonies by killing them, a conclusion that was unfortunately inaccurately drawn from our work by Nougayrède and colleagues (1). The background lawns obtained in the Ames tests indicated the absence of any cytotoxic effect by EcN, an observation that we possibly did not point out clearly enough in our publication (5). We observed a background lawn of the EcN samples that in all cases was identical to that of the H_2_O control. Thus, a possible killing effect by microcins or any other antagonistic influence of EcN on the used Salmonella Ames reporter strains can safely be excluded.

The second OECD-approved technique we used was the *in vivo* mammalian alkaline comet assay using rats, which is also recommended by the FDA (2) for detection of DNA strand breaks. In contrast, Nougayrède et al. used two alternative mouse models (1). One model utilized axenic mice that were monoassociated once with EcN and sacrificed 7 days later. Their second model used 8-day-old mouse pups that were sacrificed only 6 h after a single exposure to EcN. Colon tissue from animals of both models was assessed immunohistologically for the presence of histone γH2AX. In the rodent model we used, 7- to 9-week-old SPF rats were fed EcN daily for 2 days or 28 days. Despite this much longer exposure, histological examination and comet assay of the specified gut tissue did not detect any mutagenic effect of EcN (5). As mentioned above, the comet assay is highly appropriate in light of the proposed activity of colibactin to induce DNA strand breaks. We point out that Pharmaceutical Marketing Authorisation Holders are expected to use standardized, guideline-defined tests for comparison of obtained data. The comet assay did not display any genotoxic activity of EcN (5), but that does not make the assay “inappropriate” as expressed by Nougayrède et al. The contradictory data obtained with the different animal models can be caused by multiple factors, including the presence or absence of a matured immune system, a natural microbiota, and an intact intestinal barrier, all of which most likely reduce the direct cell-cell contact that is considered essential for the action of colibactin (6).

This is not to say that we are not concerned about the genotoxic potential of *pks*^+^ E. coli, including EcN, that has been demonstrated by alternative tests, as this potentially points toward a negative impact on human health and a role of colibactin in the development of colorectal cancer (CRC). As Nougayrède and colleagues (1) correctly stated, EcN is administered to infants. In this context, we draw attention to a recent publication describing natural mother-to-infant transmission of *pks*^+^ E. coli strains in newborns (7). Those authors concluded that “a respectable number of healthy individuals may become predisposed to a high risk of CRC at the very early stage of life”. We can confirm that *pks*^+^ E. coli bacteria are common in infants, based on as yet unpublished data. In a randomized controlled trial (unpublished data), EcN or placebo was given to newborns and again at the age of 1 year. A year later, stool samples were analyzed for the presence of *pks* by PCR, and *pks* was found in approximately 40% of infants in both groups. The incidence of *pks*^+^ E. coli in a general population has been estimated between 16% and 48% (7–9), assuming that all phylotype B2 E. coli strains contain the *pks* locus.

Numerous studies have demonstrated that CRC patients are more often colonized by phylotype B2 E. coli (e.g., reference 10), and an overrepresentation of *pks*^+^ E. coli was also demonstrated (11). A molecular signature of DNA damage that could be attributed to colibactin activity was demonstrated in whole-genome sequencing (WGS) data obtained from CRC tumors (9). However, this applied to only 5% of the investigated WGS data (9). If infants are already frequently colonized by *pks*-bearing strains, if these strains are persistent over time, and if they are able to induce colorectal cancer later in life, it needs to be explained why only such a relatively small fraction of CRC tumors bear this signature. This paradox has also been noticed by others, suggesting that the presence of commensal *pks*^+^ bacteria alone is likely insufficient for cancer development and such bacteria exert a carcinogenic influence only under specific conditions (12). The genotoxic activity of colibactin is observed only following direct cell-cell contact (6), plus the transcriptional activation of all *clb* genes of the *pks* island seems to be required (12). The direct cell-cell contact of *pks*^+^ strains with host epithelium cells is not only hampered by presence of mucus in the gut (13), but the intestinal barrier is even enhanced by EcN’s ability to promote mucus production and to seal the tight junctions (14). Transcriptional regulation of *clb* genes is influenced by many factors (e.g., metabolites), and transcription of several *clb* components is promoted by inflammation (12), but EcN is known to be anti-inflammatory by modulating the host immune system in multiple ways (14).

Beside the discussed genotoxic potential of *pks*^+^ E. coli, there are other, host-derived biological impacts in humans. Obviously, the onset of CRC is multifactorial, to which genetic factors and diet also contribute. Additionally, DNA damage repair responses play an important role (reviewed in, for example, references 12 and 15). It is therefore relevant to consider the possibility that most of the DNA damage induced by colibactin would be repaired or would generally result in apoptosis. Moreover, chronic inflammation is a known trigger of CRC. EcN reduces the risk of colitis-associated CRC, as demonstrated in ulcerative colitis (UC) patients, who maintained in remission by intake of EcN, due to its anti-inflammatory activity that is similar in potency to mesalazine (16). In addition, by means of microcin expression, EcN limits the presence of pro-inflammatory species of the family *Enterobacteriaceae* (17). Moreover, EcN did not increase microsatellite instability in UC patients (18).

It is not our intention to downplay the important findings reported by Nougayrède and coworkers (1) in any way. We just want to point out that the biological relevance of their findings, generated by *in vitro* and *in vivo* (murine) models, may not paint a complete picture of the intricate relationship between the human host and its natural gut microflora, or a microflora influenced by intake of probiotic bacteria. While we and others continue our research to elucidate this, we remain vigilant to react quickly in case evidence emerges that would suggest our EcN-based probiotic products would harm consumers.
